# Burdock (*Arctium lappa* L.) Root Extract Induces Transcriptional Signatures Associated with Metal Homeostasis and Lipid Metabolism in Human Keratinocytes

**DOI:** 10.3390/metabo16100707

**Published:** 2026-09-24

**Authors:** Cecilia Bender, Cynthia Gisela Suarez, Alejandra Mariel Habarta, Andressa Blainski, Alessandra Ruggiero, Mariano Stornaiuolo

**Affiliations:** 1 ABC-Bioresearch, 37126 Verona, Italy; alehabartabioinfo@gmail.com (A.M.H.); alessandra.ruggiero@abc-bioresearch.com (A.R.); 2 Martin Bauer Nutraceuticals Srl, 10121 Torino, Italy; cynthia.suarez-rizzo@martin-bauer.com (C.G.S.); andressa.blainski@martin-bauer.com (A.B.); 3 Finzelberg GmbH &CoKG, 56266 Andernach, Germany; 4 Department of Neurosciences, Biomedicine and Movement Sciences, School of Medicine, University of Verona, 37134 Verona, Italy; 5 Department of Pharmacy, University of Naples Federico II, 80131 Naples, Italy

**Keywords:** *Arctium lappa*, Burdock extract, skin´s lipid metabolism, sphingolipid metabolism, cholesterol homeostasis, metallothioneins, zinc homeostasis, *NRF2*, *AhR*, transcriptomics

## Abstract

**Highlights:**

**What are the main findings?**

- Burdock root extract induced an early transcriptional response in HaCaT keratinocytes characterized by activation of xenobiotic/AhR-responsive genes, metal homeostasis, and detoxification pathways, alongside lipid-, sterol-, and sphingolipid-associated transcriptional programs.
- ReactomeGSA/Camera analysis identified coordinated downregulation of mitochondrial-associated and translational gene programs together with adaptive signaling networks including RHOB/RHOC, Hippo, IL-10, and TGF-β signaling.

**What are the implications of the main findings?**

- The results suggest that Burdock root extract promotes an adaptive transcriptional state integrating redox regulation, metal homeostasis, lipid-associated responses, and epithelial remodeling pathways, including junctional and cytoskeletal remodeling, in epidermal keratinocytes.
- These findings provide transcriptomic evidence supporting the role of plant-derived bioactive compounds as modulators of metabolism-related and stress-responsive signaling networks relevant to skin health and functional botanical research.

**Abstract:**

**Background/Objectives**: Botanical metabolites can modulate cellular stress responses, lipid homeostasis, and epithelial organization. *Arctium lappa* L. (Burdock) root contains bioactive phytochemicals and inulin-type fructans, but its molecular effects in vitro remain incompletely characterized. **Methods**: HaCaT keratinocytes were exposed to a standardized aqueous Burdock root extract (1 mg/mL) for 4.5 h and analyzed by RNA sequencing. The Burdock-versus-control response was evaluated using gene-level differential-expression analysis, ReactomeGSA, and focused GO and KEGG ORA. Higher-amplitude responses were subsequently compared with those induced under identical conditions by Rosehip and Schisandra extracts. **Results**: Burdock treatment identified 80 DEGs meeting FDR ≤ 0.05 and |log_2_ FC| ≥ 1. The response was characterized by induction of metallothioneins, zinc transporters, *NRF2-* and *AhR*-associated genes. Increased expression of selected sterol-transport and intracellular-handling genes was accompanied by coordinated downregulation of cholesterol- and lanosterol-biosynthesis pathways. Reciprocal regulation of *SPHK1*, *SGPP1*, and *SGPL1* indicated altered transcriptional control of S1P production and turnover. Junctional, cytoskeletal, TGF-β-associated, and epithelial cell-state genes were also regulated. Comparative analysis identified 29 higher-amplitude DEGs meeting the predefined Burdock threshold criteria but not the corresponding comparator thresholds, including an 11-gene subgroup not significantly regulated by either comparator treatment. **Conclusions**: Together, these findings define an early transcriptional response integrating metal homeostasis, cytoprotective signaling, sterol and sphingolipid metabolism, and epithelial remodeling, providing a mechanistic framework for future investigation of Burdock rootextract nutritional and dermatological research.

## 1. Introduction

The human epidermis functions as a dynamic biological barrier continuously exposed to ultraviolet radiation, airborne particulate matter, xenobiotics, and toxic metal species. Keratinocytes, which constitute most of epidermal cells, are not merely structural components but active stress sensors that initiate adaptive transcriptional programs in response to environmental challenges. Epidermal homeostasis therefore depends on coordinated antioxidant, detoxification, inflammatory, metabolic, differentiation, and junctional pathways [1,2].

In recent years, increasing attention has been directed toward the concept of epidermal adaptation, whereby skin cells respond to environmental challenges through coordinated regulation of redox defence, lipid metabolism, micronutrient homeostasis, and intercellular barrier architecture. These adaptive processes are tightly interconnected. Alterations in cellular redox status influence lipid composition and membrane organization, while trace elements such as zinc and copper regulate enzymes involved in epithelial differentiation, wound healing, and barrier repair. Consequently, maintenance of skin health depends not only on antioxidant defences but also on the ability of keratinocytes to regulate metal availability, membrane lipid composition, and cell–cell junction integrity [1,2].

Among the cellular defence systems, nuclear factor erythroid 2-related factor 2 (NRF2, encoded by *NFE2L2*) is a master regulator of cytoprotective responses. Under basal conditions, NRF2 is sequestered in the cytoplasm by Kelch-like ECH-associated protein 1 (KEAP1), which targets NRF2 for ubiquitination and proteasomal degradation. Exposure to electrophilic compounds, oxidative stress, or reactive metabolites modifies critical cysteine residues within KEAP1, allowing NRF2 stabilization and nuclear translocation. Once in the nucleus, NRF2 binds antioxidant response elements (AREs) and activates a broad battery of genes involved in antioxidant defence, xenobiotic metabolism, glutathione synthesis, redox regulation, and metal detoxification [3,4]. Canonical NRF2 target genes include *HMOX1*, *NQO1*, *GCLC*, and members of the metallothionein family, all of which contribute to cellular resilience against oxidative and chemical stress [3,5].

An important but often underappreciated component of NRF2-mediated defence is the regulation of intracellular metal homeostasis. Metallothioneins are low-molecular-weight, cysteine-rich proteins that function as intracellular buffers for essential transition metals such as zinc and copper, sequester potentially toxic metals, and contribute to intracellular redox [6]. They also protect cells against toxic heavy metals including cadmium, mercury, and lead [6,7].

Beyond metal sequestration, metallothioneins function as potent redox modulators due to their high cysteine content, enabling direct scavenging of reactive oxygen species and maintenance of intracellular redox balance. Their expression is frequently induced by oxidative stress and electrophilic agents, and increasing evidence suggests that metallothioneins represent a critical interface between metal homeostasis and antioxidant defence mechanisms [4,6,7].

In skin, zinc availability influences DNA repair, wound healing, keratinocyte behavior, and barrier integrity [8]. Metal homeostasis may therefore operate not only as a detoxification mechanism but also as a regulator of broader epithelial metabolism. In keratinocytes, zinc availability influences multiple enzymes involved in lipid synthesis, lipid transport, and epidermal differentiation, while disruption of zinc balance has been associated with impaired barrier formation and altered membrane function. Likewise, sphingolipids, cholesterol, and free fatty acids collectively constitute the extracellular lipid matrix that is essential for stratum corneum integrity and epidermal permeability-barrier function. Regulation of cholesterol trafficking and sphingolipid metabolism therefore represents a central component of epidermal adaptation to environmental stress [9,10,11].

Cellular responses to oxidative and electrophilic stress can involve coordinated regulation of metal-buffering and lipid-remodeling systems. Metallothioneins regulate intracellular zinc availability and may thereby influence zinc-dependent signaling and metabolic processes. In parallel, S1P participates in keratinocyte proliferation, differentiation, migration, and barrier repair, whereas sterol-trafficking proteins and ATP-binding cassette transporters contribute to intracellular lipid distribution and membrane organization [8,9,10,11]. Despite these biological connections, the extent to which botanical metabolites coordinately regulate metal homeostasis and lipid-associated pathways in epidermal cells remains poorly understood.

Barrier adaptation also requires integration of metabolic regulation with intercellular junctional organization. Barrier integrity requires coordinated tight junctions, adherens junctions, and desmosomes. These structures regulate paracellular permeability and mechanical cohesion and are influenced by metabolic and environmental-sensing pathways [1,12,13,14]. Thus, barrier adaptation cannot be inferred from lipid-associated processes alone but must be considered together with junctional architecture.

In addition to NRF2, keratinocytes employ other environmental-sensing pathways that interact with redox and metabolic networks. One of the most important is AhR, a ligand-activated environmental sensor that controls xenobiotic-metabolizing enzymes, including CYP1A1 and CYP1B1, and contributes to epidermal homeostasis [15,16]. AhR and NRF2 exhibit functional crosstalk: AhR can promote *NRF2* transcription or generate reactive intermediates that engage NRF2, whereas NRF2-regulated systems process oxidative products generated during xenobiotic metabolism [16,17,18]. Coordinated regulation of these pathways may therefore connect xenobiotic metabolism with antioxidant defence and epidermal homeostasis [15,16,17,18].

Plant-derived metabolites, including phenolic acids, flavonoids, lignans, terpenoids, and oligosaccharides, can regulate redox, inflammatory, metabolic, and barrier-associated pathways [5,18]. Many phytochemicals activate NRF2-dependent signaling either directly through electrophilic interactions with KEAP1 or indirectly through modulation of cellular redox status [4,5]. Others influence membrane lipid organization, sphingolipid metabolism, inflammatory signaling, and barrier-associated processes [9,11]. Consequently, botanical extracts are widely used in dermocosmetic and nutritional formulations intended to support skin resilience and homeostasis.

*Arctium lappa* L. is an edible and medicinal plant whose root contains diverse phytochemicals, including chlorogenic (CGAs) and caffeoylquinic (CQAs)acids, arctiin, arctigenin, flavonoids, polyacetylenes, minerals, and inulin-type fructans [19,20,21,22,23]. Burdock preparations have traditionally been used in relation to skin health, wound healing, gastrointestinal function, and inflammatory conditions and have been reported to exhibit antioxidant, anti-inflammatory, antimicrobial, immunomodulatory, and metabolic activities [19,20,21,22,24]. Arctigenin and related lignans have been associated with redox-sensitive signaling, whereas inulin-type fructans contribute to the plant’s nutritional and metabolic profile [20,23]. Collectively, these observations suggest that Burdock bioactivity may arise from the combined actions of multiple classes of constituents rather than from one compound alone [19,20,22,23,24].

Despite the reported biological activities of Burdock and its chemically diverse composition, its integrated molecular effects in keratinocytes remain insufficiently characterized. For example, it is unclear whether Burdock coordinately regulates cytoprotective signaling, epithelial organization, metal homeostasis, sterol, and sphingolipid metabolism. Moreover, because botanical extracts can induce broad and overlapping stress-response programs, distinguishing shared phytochemical responses from Burdock transcriptional features remains challenging.

We hypothesized that a standardized aqueous Burdock root extract would induce an integrated early transcriptional response linking cytoprotective and xenobiotic-response pathways with metal homeostasis, lipid-associated metabolism, and epithelial remodeling. We further hypothesized that comparison with other botanical extracts evaluated under identical conditions would identify higher-amplitude transcriptional features preferentially associated with Burdock.

To assess these hypotheses, we characterized the early gene expression response of HaCaT keratinocytes to Burdock root extract. The primary objective was to define the global Burdock response and examine NRF2-, AhR-, metal-homeostasis-, lipid-metabolism-, and junction-associated transcriptional patterns. In addition, as an exploratory objective, we compared the higher-amplitude Burdock response with those induced by Rosehip and Schisandra extracts under identical experimental conditions to explore overlapping transcriptional signatures and identify candidate gene sets with distinct response profiles for Burdock root extract.

## 2. Materials and Methods

### 2.1. Botanical Extract and Quality Control

A standardized aqueous Burdock (*Arctium lappa* L.) root extract (batch TPA-39-22-1), an aqueous rosehip (*Rosa canina* L.) fruit extract (batch UB2023-54-2), and an aqueous schisandra (*Schisandra chinensis* (Turcz.) Baill.) fruit extract (batch UB2023-59-1) were supplied by Finzelberg GmbH & Co. KG (Andernach, Germany).

The Burdock extract, which represented the primary investigational material in this study, was manufactured at pilot scale for research use from 10 kg of cultivated root material sourced in Poland, using water as the sole extraction solvent. Extraction was performed by percolation at 85 °C for 5 h. The eluate was concentrated and dried to yield 3.9 kg of native dry extract (drug extract ratio [DER] 2-6:1). The botanical raw material was identified as *Arctium lappa* L. according to the relevant European Pharmacopoeia monograph for herbal medicinal materials.

The resulting extract was water-soluble preparation containing 50.1% fructans, expressed as inulin and determined by liquid chromatography with refractive index detection. Additional phytochemical characterization identified caffeoylquinic acids (1.9%) and total polyphenols (3.7%), whereas flavonoids were not detected. The extract was further evaluated for physicochemical properties, phytochemical profile, heavy metals, aflatoxins, pesticides, and microbiological quality. Detailed compositional data and analytical methods are provided in Supplementary Table S1 and Figure S1.

Rosehip and schisandra extracts were included as comparator extracts in secondary experiments and were used as supplied by the manufacturer. All extracts were stored in dry form until use.

### 2.2. Cell Culture and Treatment

HaCaT keratinocytes (CLS Cell Lines Service GmbH, Eppelheim, Germany)were maintained in high-glucose Dulbecco’s Modified Eagle Medium supplemented with 10% fetal bovine serum, 100 U/mL penicillin, and 100 µg/mL streptomycin (all fromThermo Fisher Scientific, Waltham, MA, USA) at 37 °C in a humidified 5% CO_2_ atmosphere. Immediately before cell treatment, the dry extracts were reconstituted in sterile water, sterilized by filtration through a 0.22-µm membrane filter, and diluted to the required working concentration in serum-free medium under aseptic conditions. Control cells received serum-free medium alone. Concentrations were expressed on a dry-extract basis (µg/mL).

Before transcriptomic analysis, the effects of the botanical extracts on cellular metabolic activity were evaluated across concentration range of 0.1–1 mg/mL using a resazurin-based fluorescence assay following 4.5 h of exposure. A concentration of 1 mg/mL was selected as the highest non-cytotoxic dose evaluated under these conditions, maintaining cell viability above 80% and preserving normal cell morphology throughout the exposure period.

Based on prior experimental experience with HaCaT keratinocytes, an exposure time of 4.5 h was selected to capture an established early transcriptional response while minimizing secondary effects associated with prolonged treatment. Cells were seeded in 6-well plates and incubated overnight. At approximately 80% confluence, cells were exposed to Burdock root extract for 4.5 h. Three independent biological replicates were evaluated per condition. Each replicate originated from an independently cultured and treated cell sample.

Immediately following treatment, total RNA was isolated using the RNeasy Mini Kit (Qiagen, Hilden, Germany) according to the manufacturer’s instructions. RNA samples were subsequently processed together for library preparation and sequencing. Reproducibility across biological replicates was assessed using sequencing quality-control metrics, principal component analysis (PCA), hierarchical clustering, and sample-distance analysis.

For a secondary comparative analysis, rosehip and Schisandra extracts (1 mg/mL) were evaluated under identical conditions and exposure duration (*n* = 3 per treatment). These extracts were included as exploratory botanical comparators, rather than as primary study treatments or negative controls, to differentiate transcriptional responses common across botanicals from higher-magnitude molecular features preferentially associated with burdock under matched experimental conditions.

### 2.3. RNA Extraction, Library Preparation, and Sequencing

RNA quantity and quality were assessed using an Agilent 2100 Bioanalyzer RNA assay (Agilent Technologies, Santa Clara, CA, USA). Libraries were prepared with the Universal Plus mRNA-Seq Kit (Tecan Genomics, Männedorf, Switzerland) according to the manufacturer’s instructions using the fr-secondstrand library configuration. Final libraries were evaluated with a Qubit 2.0 Fluorometer (Thermo Fisher Scientific, Carlsbad, CA, USA) and an Agilent Bioanalyzer DNA assay (Agilent Technologies, Santa Clara, CA, USA). Sequencing was performed on an Illumina NovaSeq 6000 (Illumina, Inc. Sand Diego, CA, USA) using 150bp paired-end reads. Burdock libraries generated 124.52, 144.18, and 141.83 million reads, and untreated-control libraries generated 156.50, 129.21, and 136.62 million reads, corresponding to mean depths of 136.84 and 140.78 million reads per sample, respectively.

### 2.4. Bioinformatic and Differential-Expression Analysis

Base calling, demultiplexing, and adapter masking were performed using Illumina BCL Convert v3.9.3. Adapter 6000 (Illumina, Inc. Sand Diego, CA, USA) sequences were masked during demultiplexing, and low-quality bases and residual adapter sequences were removed during sequence preprocessing. The resulting high-quality reads aligned to the human reference genome and transcriptome (hg38, iGenomes) using STAR with default parameters [25]. Transcript assembly and gene-level quantification were performed using StringTie with default parameters [26]. RNA-seq quality-control metrics were performed using RSeQC [27] and included assessment of library strandness, gene-body coverage, read distribution, and insert-size distribution. FastQC, STAR, and RSeQC results were aggregated in MultiQC. All libraries passed the applied sequencing and alignment quality-control criteria and were retained for downstream analysis.

Gene- and transcript-level differential-expression analyses were performed using DESeq2 [28,29], with three independent biological replicates per condition. Pairwise comparisons were conducted by fitting a generalized linear model to the count data for each feature. Count normalization was performed using the median-of-ratios method, and statistical significance was evaluated using the Wald test. DESeq2 shrinkage estimation was applied to dispersion and fold-change estimates to improve their stability and interpretability. Raw *p*-values were adjusted for multiple testing using the Benjamini–Hochberg procedure to control the false discovery rate (FDR). Genes with FDR ≤ 0.05 were considered differentially expressed. Variance-stabilizing transformation (VST) was applied to normalized gene-count data for PCA, hierarchical clustering, and sample-distance evaluation.

### 2.5. Functional Enrichment and GeneSet

A complementary directional gene-set analysis was performed using the ReactomeGSA web interface [30], implementing the CAMERA algorithm [31] (Reactome version 97). The complete VST-transformed expression matrix generated via DESeq2 (Section 2.4), encompassing all evaluated genes without applying a preliminary FDR cutoff, was analyzed across Burdock extract-treated and control replicates (*n* = 3 per group) against curated human biological pathways. Pathways with an adjusted *p*-value (FDR) < 0.05 were considered significantly regulated. Pathway direction was assigned by CAMERA [31], and average pathway log _2_“fold-change” (log_2_ FC) represented the mean expression difference across all genes mapped to a given pathway.

Over-Representation Analysis (ORA) for Gene Ontology (GO) Biological Process terms and KEGG pathways was performed specifically on the 80 high-amplitude DEGs (59 upregulated and 21 downregulated) identified using high stringency thresholds in DESeq2 (Section 2.4). ORA was implemented using the enrichGO and enrichKEGG functions of the clusterProfiler R package [32,33], based on a hypergeometric test with Benjamini–Hochberg correction (FDR < 0.05). In enrichment plots, GeneRatio represented the proportion of input high-amplitude DEGs assigned to a given pathway, whereas point size reflected the total number of mapped genes associated with that pathway.

### 2.6. Cellular Antioxidant Activity (CAA)

CAA was determined in HaCaT keratinocytes as previously described [34,35]. Cells were incubated with six non-cytotoxic concentrations of *Arctium lappa* root extract (batch TPA 39-22-1) together with a cell-permeable fluorescent probe for 60 min at 37 °C. Following washing, a free radical initiator was added, and fluorescence was monitored kinetically for 1 h. Antioxidant activity was quantified using a quercetin calibration curve and expressed as μmol of quercetin equivalents (QE) per g of extract. Commercial inulin (TPA 29-23) was included as a comparator and evaluated on an equivalent inulin-content basis.

## 3. Results

### 3.1. Global Transcriptomic Response

Burdockextract treated and untreated-control libraries displayed comparable sequencing depths and consistently high alignment quality. All six libraries passed the applied sequencing and alignment quality-control criteria and were retained for downstream analysis. Sequencing, alignment, and differential-expression results for the Burdock-versus-control comparison are summarized in Table 1.

PCA of VST expression values for the 500 most variable genes showed clear separation between the three Burdock-treated replicates and the three untreated controls. PC1 and PC2 accounted for 84.0% and 8.5% of the total variance, respectively (Figure 1A).

The volcano plot summarizes the distribution of effect sizes and statistical significance across the Burdock-versus-control comparison. Genes meeting both FDR ≤ 0.05 and |log_2_ FC| ≥ 1 are distinguished from genes meeting the FDR criterion alone (Figure 1B). Hierarchical clustering of the 50 most variable significant DEGs separated the Burdock-treated and untreated-control samples into distinct groups (Figure 1C).

DESeq2 analysis identified a broad gene-level transcriptional response to Burdock treatment. The complete set of significant DEGs was used for the primary gene-level assessment, whereas the additional |log_2_ FC| threshold defined the higher-amplitude DEG set used for focused ORA and comparative analyses. The corresponding DEG counts and direction of regulation are reported in Table 1. Sample-level relationships across the complete experimental dataset were additionally evaluated using a sample-distance matrix calculated from VST expression values (Figure S2). Collectively, PCA, differential-expression analysis, and hierarchical clustering showed a consistent separation between Burdock-treated and untreated-control samples (Figure 1A–C).

### 3.2. Functional Enrichment and Directional Pathway Analysis

Functional enrichment was examined using two complementary approaches. ReactomeGSA analysis evaluated coordinated directional changes across the complete expression dataset, whereas focused GO and KEGG ORA were performed using the 80 higher-amplitude DEGs. ReactomeGSA analysis included 12,967 mapped genes and 2762 tested pathways. Among these, 190 pathways met FDR ≤ 0.05, comprising 44 pathways with positive and 146 pathways with negative average directions.

Pathways with significant positive average directions included the RHOB and RHOC GTPase cycles, signaling by Hippo, metallothioneins bind metals, response to metal ions, and RUNX1 regulation of tight-junction components. Metallothioneins bind metals included eight mapped genes and showed an average pathway log_2_ FCof +0.784 at FDR = 0.0022. Response to metal ions included 11 genes and showed an average pathway log_2_ FC of +0.578 at FDR = 0.0067. Signaling by Hippo included 20 genes and showed an average pathway log_2_ FC of +0.178 at FDR = 7.8 × 10^−4^, whereas RUNX1 regulation of tight-junction components included four genes and showed an average pathway log_2_ FC of +0.372 at FDR = 0.0162 (Figure 2A).

Pathways with significant negative average directions included translation, mitochondrial translation, respiratory electron transport, Complex I biogenesis, ribosome-associated quality control, and protein targeting to membranes. Respiratory electron transport included 143 genes and showed an average pathway log_2_ FCof −0.161 at FDR = 3.57 × 10^−14^. Mitochondrial translation included 97 genes and showed an average pathway log_2_ FC of −0.149 at FDR = 1.80 × 10^−11^, whereas translation included 332 genes and showed an average pathway log_2_ FC of −0.119 at FDR = 3.06 × 10^−11^ (Figure 2A).

Sterol-biosynthetic pathways also showed significant negative average directions. Lanosterol biosynthesis included 15 mapped genes and showed an average pathway log_2_ FC of −0.278 at FDR = 2.6 × 10^−5^. Cholesterol biosynthesis included 28 genes and showed an average pathway log_2_ FC of −0.188 at FDR = 3.7 × 10^−5^. Cholesterol biosynthesis via desmosterol included 10 genes and showed an average pathway log_2_ FC of −0.146 at FDR = 0.0376. Consistent gene-level decreases were observed for *HMGCS1*, *MVD*, *IDI1*, *FDPS*, *LSS*, *TM7SF2*, and *HSD17B7*.

Focused GO Biological Process ORA of the 80 higher-amplitude DEGs identified enrichment of terms associated with responses to cadmium, copper, zinc, and metal ions; intracellular zinc-ion homeostasis; cellular responses to metal ions; and detoxification of inorganic compounds (Figure 2B). These terms included *MT1G*, *MT1X*, *MT1F*, *MT1E*, and *HMOX1* among the mapped genes. Coagulation- and hemostasis-associated terms were also enriched and included *F3*, *SERPINE1*, *PLAU*, and *F2R* among the mapped genes.

KEGG ORA of the same 80-gene set identified significant enrichment of TGF-β signaling, mineral absorption, signaling pathways regulating pluripotency of stem cells, hippo signaling, AGE-RAGE signaling in diabetic complications, complement and coagulation cascades, and tryptophan metabolism (Figure 2C). TGF-β signaling showed the highest GeneRatio among the represented pathways.

Genes mapped to TGF-β signaling included *TGFB2*, *PMEPA1*, *SERPINE1*, *F3*, TGF-β receptor genes, and SMAD-associated transcripts. Genes mapped to complement and coagulation cascades included *F3*, *SERPINE1*, *PLAU*, and *F2R*.

ReactomeGSA pathways associated with PPARα-responsive transcription and sphingolipid metabolism showed positive average directions but did not meet FDR ≤ 0.05. By contrast, the negative direction of cholesterol-biosynthesis pathways met the FDR criterion and was accompanied by decreases in multiple sterol-biosynthetic genes.

### 3.3. NRF2- and Metal-Homeostasis-Associated Response

DESeq2 analysis showed a modest increase in *NFE2L2* expression (log_2_ FC= +0.25), accompanied by significant increases in NRF2-associated antioxidant and detoxification genes, including *HMOX1* (log_2_ FC= +1.06), *GCLC* (log_2_ FC= +0.78), *GCLM* (log_2_ FC= +0.37), and *TXNRD1* (log_2_ FC= +0.71). Metallothionein genes were also upregulated, including *MT1G* (log_2_ FC= +5.64), *MT1X* (log_2_ FC= +2.00), *MT1F* (log_2_ FC= +1.62), *MT1E* (log_2_ FC= +1.16), and *MT2A* (log_2_ FC= +0.90). The zinc-transporter genes *SLC30A1* (log_2_ FC= +1.30) and *SLC39A10* (log_2_ FC= +1.07) were similarly increased. These gene-level findings were consistent with the ReactomeGSA and focused on GO results for metallothionein and metal-ion-associated pathways.

In a complementary functional assay performed in HaCaT keratinocytes, the standardized *Arctium lappa* root extract exhibited measurable cellular antioxidant activity, with a CAA value of 23.36 μmol QE/g extract. Commercial inulin tested on an equivalent inulin-content basis showed a lower CAA value of 17.54 μmol QE/g.

### 3.4. AhR-Associated Xenobiotic Response

DESeq2 analysis identified significant increases in AhR-associated genes, including *CYP1A1* (log_2_ FC= +1.96), *CYP1A2* (log_2_ FC= +1.70), *CYP1B1* (log_2_ FC= +1.12), *AHRR* (log_2_ FC= +0.96), *ALDH3A1* (log_2_ FC= +0.79), *TIPARP* (log_2_ FC= +0.40), and *AHR* (log_2_ FC= +0.31). The concurrent increases in cytochrome P450 genes and AhR-associated regulatory genes defined a coordinated AhR-associated transcriptional response.

### 3.5. Lipid and Sphingolipid Remodeling

DESeq2 analysis identified significant changes in genes associated with sterol transport, cholesterol biosynthesis, retinoid metabolism, and S1P metabolism (Figure 3A).

Among the transport-associated genes, *ABCG1* (log_2_ FC= +1.96), *ABCG2* (log_2_ FC= +0.65), *OSBP2* (log_2_ FC= +1.44), and *OSBP* (log_2_ FC= +0.37) were significantly increased. In contrast, many key genes associated with cholesterol biosynthesis were decreased, including *HMGCS1* (log_2_ FC= −0.65), *FDPS* (log_2_ FC= −0.47), and *LSS* (log_2_ FC= −0.45). Other sterol-biosynthetic genes, including *MVD*, *IDI1*, *TM7SF2*, and *HSD17B7*, also contributed to the significant negative direction of the corresponding ReactomeGSA pathways. *CYP26A1* (log_2_ FC= −1.38), which is associated with retinoic acid catabolism, was also downregulated.

Within the S1P metabolic network, *SPHK1* was downregulated (log_2_ FC= −0.97), whereas *SGPP1* (log_2_ FC= +1.01) and *SGPL1* (log_2_ FC= +0.54) were upregulated. Thus, the three principal gene-level changes showed coordinated regulation of S1P synthesis, dephosphorylation, and degradation.

At the gene level, the lipid-associated response therefore comprised increased expression of selected transport-associated genes, decreased expression of multiple sterol-biosynthetic genes, and coordinated changes in genes involved in S1P metabolism.

### 3.6. Junctional and Barrier-Associated Remodeling

DESeq2 analysis identified moderate but coordinated increases in genes associated with tight junctions, desmosomes, and adherens junctions (Figure 3B). Tight-junction-associated genes included *CLDN1* (log_2_ FC= +0.78), *CLDN16* (log_2_ FC= +0.91), *OCLN* (log_2_ FC= +0.62), *TJP1* (log_2_ FC= +0.50), and *TJP2* (log_2_ FC= +0.26). Desmosomal genes included *DSG2* (log_2_ FC= +0.65), *DSG3* (log_2_ FC= +0.77), *DSG4* (log_2_ FC= +1.27), *DSC2* (log_2_ FC= +0.62), *DSC3* (log_2_ FC= +0.63), *DSP* (log_2_ FC= +0.52), and *JUP* (log_2_ FC= +0.27). The adherens-junction gene *CDH1* was also increased (log_2_ FC= +0.67).

Among these junction-associated genes, only *DSG4* met |log_2_ FC| ≥ 1. The remaining genes showed significant but lower amplitude increases.

### 3.7. Differentiation-Associated Remodeling

DESeq2 analysis identified decreased expression of *ID1* (log_2_ FC= −1.40), *ID2* (log_2_ FC= −3.73), *ID3* (log_2_ FC= −2.22), *ID4* (log_2_ FC= −2.67), *DLX2* (log_2_ FC= −1.21), *DLX5* (log_2_ FC= −1.29), *DLX6* (log_2_ FC= −1.32), and *HEY1* (log_2_ FC= −1.14). Among the additional epithelial-state-associated genes examined, *IVL* was decreased (log_2_ FC= −0.58), whereas *KRT17* was increased (log_2_ FC= +0.40). The observed expression pattern therefore comprised repression of multiple *ID* and *DLX* family genes without a concurrent increase in *IVL*.

### 3.8. Burdock Threshold Defined Higher-Amplitude Signature

To compare higher-amplitude transcriptional responses across extracts, differential-expression results from the Burdock-, Rosehip-, and Schisandra-versus-control contrasts were filtered using the same predefined criteria of FDR ≤ 0.05 and |log_2_ FC| ≥ 1. Genes meeting both criteria in the Burdock-versus-control contrast but not meeting both criteria in either comparator-versus-control contrast were classified as Burdock threshold-defined higher-amplitude genes. Of note, this classification describes differences based on the applied statistical and effect-size thresholds and does not establish statistically significant differences between treatment effects.

The 80 higher-amplitude Burdock-responsive genes were then compared with the correspondingly filtered Rosehip and Schisandra datasets. Of the 80 higher-amplitude Burdock-responsive genes, 29 did not simultaneously meet FDR ≤ 0.05 and |log_2_ FC| ≥ 1 in either comparator analysis (Figure 4). The comparison also identified seven genes shared by Burdock and Schisandra, 23 shared by Burdock and Rosehip, and 21 shared by all three treatments.

Classification was based on the combined statistical significance and effect-size thresholds. Consequently, some genes included in the 29-gene Burdock preferential signature showed statistically significant but lower-amplitude regulation following Rosehip or Schisandra treatment. These genes met both stringent criteria following Burdock treatment but did not simultaneously reach FDR ≤ 0.05 and |log_2_ FC| ≥ 1 in either comparator dataset.

The 29-gene signature included *ITPR1*, *SGPP1*, and *OSBP2*. *ITPR1* showed log_2_ FC= +1.369 and FDR = 1.10 × 10^−4^, whereas *SGPP1* showed log_2_ FC= +1.012. *OSBP2* showed log_2_ FC= +1.439 and FDR = 1.61 × 10^−23^ and was not significant in either comparator analysis.

Within the 29-gene signature, 11 genes had FDR > 0.05 in both comparator analyses (Table 2), of which 10 were upregulated following Burdock treatment.

The subgroup included genes associated with metal handling (*MT1G*, *MT1F*, and *SCARA5*), sterol transport (*OSBP2*), TGF-β-associated responses (*PMEPA1* and *TGFB2*), and coagulation-associated signaling (*F3*). Within this subgroup, *MT1G* showed the greatest effect magnitude, followed by *PMEPA1*, *MT1F*, *SCARA5*, and *OSBP2*.

## 4. Discussion

The present study combined gene-level differential-expression analysis, transcriptome-wide directional pathway analysis, focused ORA, and comparison with two botanical extracts to characterize the early response of HaCaT keratinocytes to Burdock root extract. The results revealed an integrated transcriptional program involving NRF2- and AhR-associated responses, metallothionein-mediated metal homeostasis, sterol, and sphingolipid metabolism, and epithelial remodeling. The correspondence between gene-level and pathway-level results was particularly evident for metal homeostasis and sterol biosynthesis, whereas the comparative analysis identified a smaller set of responses expressed with greater amplitude, or detected only, following Burdock rootextract treatment.

### 4.1. Integrated Cytoprotective and Metal-Homeostasis Response

The increase in *NFE2L2* expression, together with the induction of *HMOX1*, *GCLC*, *GCLM*, *TXNRD1*, and several metallothionein genes, is consistent with an NRF2-associated cytoprotective response. NRF2 coordinates cellular adaptation to electrophilic and oxidative stress by regulating antioxidant defence, glutathione synthesis, redox balance, xenobiotic metabolism, and metal-responsive genes [3,4,5]. Consistent with this interpretation, the Burdock root extract exhibited measurable intracellular antioxidant activity in HaCaT keratinocytes. In the CAA assay, the extract showed greater antioxidant activity than commercial inulin tested on an equivalent inulin-content basis. This finding suggests that constituents other than inulin, or interactions among multiple constituents within the extract, contributed to the observed cellular antioxidant activity. Although consistent with the activation of cytoprotective pathways, these data do not establish which extract components or cellular mechanisms are primarily responsible for the observed effect.

In parallel, increased expression of *CYP1A1*, *CYP1A2*, *CYP1B1*, *AHRR*, *TIPARP*, and *ALDH3A1* supports engagement of an AhR-associated transcriptional program. AhR detects structurally diverse environmental and dietary compounds and regulates xenobiotic-metabolizing enzymes, whereas *AHRR* and *TIPARP* contribute to negative-feedback regulation of the pathway. Functional crosstalk between AhR and NRF2 allows xenobiotic metabolism and antioxidant defence to be coordinated during cellular adaptation [15,16,17,18]. The Burdock response should therefore not be interpreted as the isolated activation of one receptor pathway, but as the simultaneous transcriptional engagement of complementary environmental-response systems.

A particularly prominent component was the induction of *MT1G*, *MT1X*, *MT1F*, *MT1E*, and *MT2A*. Metallothioneins are small, cysteine-rich proteins capable of binding zinc, copper, and potentially toxic metal ions. Their thiol-rich structure also allows them to participate in redox regulation. Rather than functioning simply as static metal-storage proteins, metallothioneins can buffer intracellular metal availability and release or redistribute zinc in response to redox and metabolic signals [6,7,36].

The concomitant induction of *SLC30A1* and *SLC39A10* further connect the metallothionein response with zinc homeostasis. Members of the SLC30 family reduce cytosolic zinc by promoting zinc export or sequestration into intracellular compartments, whereas SLC39 transporters increase cytosolic zinc by facilitating its entry from extra or intracellular stores. Coordinated regulation of these systems could therefore allow keratinocytes to control the availability of zinc for signaling and zinc-dependent cellular processes while limiting metal-associated stress [8,36].

The prominence of this response was supported by both analytical levels. ReactomeGSA identified positive regulation of the human pathways “Metallothioneins bind metals” and “Response to metal ions” whereas focused GO ORA highlighted “Responses to zinc, copper, cadmium, and inorganic compounds.” The cadmium-related annotations likely arise as metallothioneins have established roles in cellular responses to several metal ions, including cadmium, zinc, and copper. Experimental studies in human keratinocytes have similarly associated metal-responsive stress with metallothionein induction and altered zinc and copper homeostasis [37,38].

Furthermore, the comparative analysis reinforced the importance of this module. *MT1G* and *MT1F* belonged to the 11-gene subgroup that was not significantly regulated by either comparator treatment under the experimental conditions, while *MT1G* showed the largest effect magnitude within that 11-gene subgroup.

Additionally, *SCARA5*, which encodes a scavenger receptor associated with ferritin recognition and cellular iron handling, added an iron-related component.

Collectively, these observations identify metal buffering and metal availability as prominent features of the early Burdock response rather than secondary consequences of a general antioxidant program.

### 4.2. Transcriptional Modulation of Sterol, Sphingolipid, and Lipid-Associated Pathways

#### 4.2.1. Cholesterol Transport and Intracellular Sterol Handling

The lipid-associated response was characterized by opposing transcriptional directions for sterol transport and sterol biosynthesis. Increased expression of *ABCG1*, *OSBP2*, and *OSBP* indicated greater transcriptional representation of cholesterol transport and intracellular sterol-handling functions. By contrast, multiple genes of the sterol-biosynthetic pathway were downregulated. This pattern is consistent with a remodeling of sterol homeostasis rather than uniform stimulation or suppression of lipid-associated processes.

Among the lipid-associated genes, *ABCG1*—encoding an ATP-dependent transporter involved in cellular sterol handling and cholesterol export—exhibited one of the largest transcriptional inductions. While functional cholesterol efflux depends on downstream protein translation and extracellular acceptors, the marked upregulation of *ABCG1* mRNA points to an enhanced transcriptional priming for sterol export machinery. A relevant mechanistic precedent exists in THP-1 macrophages, where arctigenin, a key bioactive lignan of *Arctium lappa*, upregulated *ABCA1*, *ABCG1*, and *APOE* expression via PPARγ/LXRα signaling [39]. Although that study evaluated a purified constituent in an immune cell model, it confirms that Burdock-derived compounds can target the transcriptional networks controlling cellular cholesterol distribution, providing a solid framework for the *ABCG1* induction observed in our keratinocyte model.

Among the 11-gene subgroup exhibiting distinct response profiles relative to the comparator extracts, *OSBP2* was particularly notable. *OSBP2* encodes oxysterol-binding protein 2, a member of a protein family operating at membrane-contact sites to facilitate sterol and phospholipid exchange across cellular membranes, thereby influencing membrane composition, organelle communication, and lipid-sensitive signaling [40]. The selective upregulation of *OSBP2* mRNA highlights intracellular sterol handling as a prominent lipid-associated feature characterizing the early Burdock root extract transcriptional response.

Conversely, the induction of *ABCG2* should be interpreted in a distinct context. Unlike *ABCG1*, *ABCG2* encodes a broad-substrate efflux transporter involved in the cellular export of xenobiotics and endogenous metabolites, whose transport activity is strongly influenced by the lipid composition of the surrounding membrane. Rather than serving as a direct mechanism for cholesterol efflux, increased *ABCG2* expression likely links the lipid-associated transcriptional response to the broader AhR-mediated xenobiotic-defence program.

#### 4.2.2. Coordinated Repression of Sterol Biosynthesis

Increased expression of selected sterol-transport and intracellular-handling genes occurred alongside reduced expression of *HMGCS1*, *MVD*, *IDI1*, *FDPS*, *LSS*, *TM7SF2*, and *HSD17B7*. These genes act at successive stages of the mevalonate and sterol-biosynthetic pathways. HMGCS1 contributes to the formation of HMG-CoA, MVD and IDI1 participate in the generation and interconversion of isoprenoid precursors, FDPS produces farnesyl pyrophosphate, and LSS catalyzes the cyclization of oxidosqualene to lanosterol. TM7SF2 and HSD17B7 contribute to subsequent downstream reactions in sterol synthesis.

Although individual transcript changes were modest in magnitude, ReactomeGSA independently identified significant negative regulation across key sterol pathways, including lanosterol biosynthesis, cholesterol biosynthesis, and the desmosterol branch of cholesterol synthesis. This pathway-level concordance demonstrates that the observed suppression was not driven by a single isolated gene, but rather reflects a coordinated, multi-node downregulation across the entire biosynthetic pathway.

The combined expression pattern is therefore consistent with reduced transcriptional investment in de novo sterol biosynthesis accompanied by increased expression of selected genes involved in sterol transport and intracellular distribution. This redistribution model is biologically distinct from generalized lipid accumulation or non-specific suppression of lipid metabolism, suggesting that keratinocytes adjust how sterols are transported and allocated among membranes while reducing the transcriptional demand for their synthesis.

While single-constituent literature on arctigenin provides a helpful framework for sterol-efflux targets [39], isolated-compound studies do not capture the broad, multi-node biosynthetic shifts identified here. Consequently, the coordinated downregulation of cholesterol-biosynthesis pathways represents an integrated feature of the complete Burdock root extract response, underscoring the functional complexity captured when evaluating the whole botanical matrix.

#### 4.2.3. S1P Metabolism and Sphingolipid Recycling

The sphingolipid-associated response was more targeted than the sterol response. While the broad Reactome sphingolipid metabolism pathway did not reach statistical significance at the pathway level (FDR ≥ 0.05), three functionally connected genes exhibited reciprocal regulation: *SPHK1* was downregulated, whereas *SGPP1* and *SGPL1* were upregulated. These genes control crucial sequential steps in sphingosine-1-phosphate (S1P) turnover. *SPHK1* phosphorylates sphingosine to generate S1P. *SGPP1* reverses this reaction by dephosphorylating S1P back to sphingosine, whereas *SGPL1* irreversibly cleaves S1P, permanently removing it from the sphingolipid pool. Their coordinated regulation points to a targeted transcriptional remodeling of S1P turnover.

*SGPP1* is of biological interest because the sphingosine generated via S1P dephosphorylation can re-enter the sphingolipid salvage pathway for reacylation by ceramide synthases, recycling sphingoid bases into ceramides and complex sphingolipids [41]. Increased *SGPP1* mRNA expression may therefore favor the recovery of sphingosine for ceramide synthesis, whereas elevated *SGPL1* favors irreversible S1P catabolism. The functional outcome of this transcriptional shift would depend on post-transcriptional enzyme kinetics, substrate availability, and net metabolic flux.

The physiological relevance of S1P turnover in epidermal biology is well established. Disruption of *Sgpp1* in mouse keratinocytes severely alters intracellular S1P levels, epidermal architecture, and differentiation marker expression [42]. Beyond structural barrier integrity, S1P signaling and transport modulate keratinocyte proliferation, differentiation, migration, inflammatory crosstalk, and epithelial stress responses [11,43,44]. Furthermore, cellular S1P clearance and recycling are dynamically regulated by specific lipid phosphatases, reflecting a tight connection between extracellular signaling cues and intracellular sphingolipid handling [45].

Comparative analyses refine this interpretation. *SGPP1* was significantly induced across all three botanical treatments, but only Burdock exceeded the high-amplitude threshold (|log_2_ FC| ≥ 1). *SGPP1* thus represents a shared botanical response expressed with higher magnitude following Burdock exposure, rather than a unique feature. In contrast, *OSBP2* was not significantly regulated by Rosehip or Schisandra. The lipid-associated comparative signature consequently combines a selectively detected sterol-handling component (*OSBP2*) with an amplified, shared sphingolipid-turnover response (*SGPP1*).

#### 4.2.4. Integration with Retinoid Metabolism

The downregulation of *CYP26A1* provides an additional mechanistic connection to retinoid handling. *CYP26A1* encodes an endoplasmic reticulum cytochrome P450 enzyme that catalyzes the hydroxylation and subsequent inactivation of all-trans-retinoic acid (atRA), thereby serving as a major regulator of intracellular retinoic acid homeostasis [46]. Retinoid signaling strongly modulates epidermal proliferation, differentiation programs, and tissue remodeling. Reduced *CYP26A1* expression could therefore diminish retinoic acid breakdown, representing a transcriptional mechanism to modulate localized retinoid sensitivity. This response acts adjacent to, yet mechanistically distinct from, primary sterol and sphingolipid pathways.

Together, the findings in Section 4.2 define a coherent transcriptional program: increased expression of key sterol-transport genes (*ABCG1*, *OSBP2*), coordinated suppression of de novo sterol synthesis (*HMGCS1*, *LSS*), reciprocal tuning of S1P-metabolizing enzymes (*SGPP1*, *SGPL1*, *SPHK1*), and attenuation of retinoid catabolism (*CYP26A1*). Because sterols, sphingolipids, and retinoid signals collectively govern epidermal membrane dynamics and permeability-barrier homeostasis, this combined response provides a plausible metabolic bridge connecting cellular stress adaptation to broader epithelial remodeling [9,10,11].

The present study was designed to characterize the response to the whole standardized aqueous Burdock root extract rather than to attribute its effects to individual constituents. The coordinated regulation of cytoprotective, metal-homeostasis, sterol-, sphingolipid-, and epithelial-remodeling-associated pathways may therefore reflect the activity of the complete botanical matrix. Although combined or interactive effects among its constituents are possible, pharmacological synergy cannot be established from the present whole-extract study.

### 4.3. Calcium, TGF-β, and Pericellular-Response Signaling

The 29-gene high-amplitude signature with distinct Burdock response profiles contained *ITPR1*, which encodes an IP_3_-gated calcium channel located in the endoplasmic reticulum (ER). Following phospholipase C-dependent production of IP_3_, ITPR1 releases Ca^2+^ from intracellular stores, contributing to spatially and temporally regulated calcium signals that govern keratinocyte adhesion, migration, differentiation, and re-epithelialization [47,48]. The co-occurrence of *ITPR1*, *SGPP1*, and *OSBP2* within this signature suggests a potential intersection between ER calcium signaling and lipid-regulatory pathways involving sphingolipid and sterol metabolism. Because calcium and lipid homeostasis are frequently coordinated at ER-associated membrane-contact sites, this observation raises the possibility of a functionally integrated signaling module. However, co-expression alone does not establish direct molecular interaction; thus, this interpretation should remain hypothesis-generating pending future functional validation.

The comparative dataset also featured *TGFB2*, *PMEPA1*, *SERPINE1*, and *F3;* with *TGFB2*, *PMEPA1*, and *F3* belonging to the 11-gene subgroup that was not significantly regulated by either comparator extract. TGF-β signaling regulates keratinocyte proliferation, migration, re-epithelialization, inflammatory crosstalk, and extracellular matrix (ECM) organization, though its net biological outcome depends on ligand concentration, exposure duration, cell state, and pathway crosstalk [49,50]. The concurrent induction of *TGFB2* and *PMEPA1* is particularly informative because these genes represent components associated with both activation and feedback regulation of the TGF-β signaling network. *TGFB2* encodes a secreted ligand, whereas *PMEPA1* encodes a transmembrane regulator that attenuates canonical SMAD signaling through negative-feedback mechanisms [51]. Their simultaneous induction indicates active engagement of TGF-β-associated regulatory loops during early cellular adaptation to Burdock exposure.

*SERPINE1* and *F3* extend this response toward pericellular proteolysis and cell-surface regulation. *SERPINE1* (encoding PAI-1) inhibits plasminogen activators, modulating pericellular matrix turnover, cell adhesion, and cell motility. *F3* encodes tissue factor, which, beyond its classic role in extrinsic coagulation, participates in signaling pathways that promote inflammatory responses, protease-activated receptor (PAR) signaling, and tissue remodeling. Their co-regulation explains the enrichment of coagulation- and hemostasis-associated pathways in our functional annotations; in this epithelial context, such terms reflect shared protease-regulatory machinery and cell-surface interaction networks rather than systemic blood clotting.

Finally, *LMO7* and *RGMA* extend this pericellular program to cytoskeletal and cell-surface architecture. LMO7 localizes to cell–cell junctions and cortical actin structures, maintaining epithelial integrity under mechanical or osmotic stress [52]. RGMA is a cell-surface guidance molecule with reported roles in cell migration and BMP-associated signaling that links neogenin-associated pathways with bone morphogenetic protein (BMP) regulation [53]. Together, these findings are consistent with an early pericellular-response program incorporating ER calcium dynamics, growth-factor regulatory loops, pericellular proteolysis, and structural cytoskeletal remodeling.

### 4.4. Junctional and Cell-State Remodeling

The coordinated induction of tight-junction, adherens-junction, and desmosomal genes points to a comprehensive transcriptional remodeling of keratinocyte junctional architecture. Upregulation of *CLDN1*, *CLDN16*, *OCLN*, *TJP1*, and *TJP2* involved key structural and regulatory components of tight-junction complexes, whereas increased *CDH1* expression was consistent with enhanced adherens-junction organization. Concurrently, elevated expression of desmosomal cadherins (*DSG2*, *DSG3*, *DSG4*, *DSC2*, *DSC3*) and plaque components (*DSP*, *JUP*) extended this structural response to mechanical cohesion across the cell layer. While tight junctions regulate paracellular solute and ion permeability, adherens junctions, and desmosomes couple neighboring cells structurally and distribute mechanical forces throughout epithelial sheets [12,13,14].

Functional enrichment analysis provided robust network-level context for this structural response. ReactomeGSA identified positive regulation of RHOB and RHOC GTPase cycles, Hippo signaling, and RUNX1-mediated regulation of tight-junction components, with KEGG ORA independently confirming Hippo pathway enrichment. Rho-family GTPases coordinate membrane-associated signals with actin dynamics, cell migration, and junction assembly [54], whereas Hippo signaling integrates cell–cell contact, cell polarity, and cytoskeletal tension to regulate tissue architecture and cell fate [55]. The tight alignment between these enriched signaling cascades and the junctional DEGs is consistent with coordinated structural reinforcement and mechanotransduction-related responses. Moreover, enrichment of IL-10 signaling suggests the involvement of regulatory pathways associated with epithelial homeostasis, indicating effects that extend beyond classical xenobiotic and metabolic stress responses. Rather than inducing uniform terminal differentiation, the gene expression profile reflects a subtle, non-canonical remodeling of cell state. Transcriptional repressors and developmental regulators including *ID1*, *ID2*, *ID3*, *ID4*, *DLX2*, *DLX5*, *DLX6*, and *HEY1* were markedly downregulated, suggesting substantial reprogramming of transcriptional networks involved in cell-fate control. However, *IVL*, a classic marker of cornified-envelope formation, was also decreased, whereas the stress- and activation-associated keratin *KRT17* was upregulated. Furthermore, induction of *JUN*, which encodes a core subunit of the AP-1 transcription factor complex, points to active regulation of keratinocyte stress adaptation, proliferation, and early tissue remodeling [56]. Consequently, these changes do not signal simple lineage maturation. A more consistent interpretation is the induction of an activated, adaptive epithelial state characterized by junctional and cytoskeletal reinforcement occurring alongside the selective reprogramming of cell-state regulators.

Additional transcripts within the comparative signature broaden this adaptive response toward cell-surface interaction, regulatory crosstalk, and growth modulation. *B3GNT7* encodes a glycosyltransferase that modifies cell-surface glycans, potentially altering membrane protein interactions with neighboring cells and the extracellular matrix. Concurrently, regulation of *IGFBP3* (modulating IGF availability), *APCDD1* (a membrane-bound Wnt signaling inhibitor), the non-coding RNA *SCARNA2*, and the intracellular cation channel *TMEM176B* points to modulation of signaling, growth-regulatory, and ion-transport processes [57,58,59]. While their precise individual roles in the Burdock response warrant further study, their collective induction is consistent with broader modulation of cell-surface signaling and growth-regulatory pathways.

Finally, ReactomeGSA identified coordinated negative regulation of basal translational machinery, mitochondrial translation, respiratory electron transport, Complex I biogenesis, and protein targeting to membranes. Because these pathway-level shifts were mapped across the entire expressed transcriptome, they reflect a clear early reduction in the transcriptional representation of protein synthesis and mitochondrial bioenergetics. Mitochondrial respiratory capacity is tightly regulated by nutrient availability and cellular stress cues [60], and bioenergetic rewiring is fundamentally linked to keratinocyte state transitions [61]. In the present study, the suppression of bioenergetic pathways occurred concurrently with the selective induction of metal-handling, lipid-transport, and junctional genes. This inverse pattern is consistent with a re-allocation of early transcriptional resources toward epithelial barrier reinforcement and stress adaptation rather than a state of non-specific transcriptional shutdown.

### 4.5. Integrated Transcriptomic Response to Burdock Root Extract

Collectively, our transcriptomic findings demonstrate that early exposure to Burdock root extract elicits a highly coordinated cellular adaptation program in HaCaT keratinocytes.

This response is organized into five major upregulated transcriptional modules: (1) AhR-mediated xenobiotic-defence networks, (2) sterol transport and intracellular sterol handling, (3) targeted sphingolipid turnover and S1P recycling, (4) heavy-metal buffering and zinc homeostasis, and (5) adaptive pericellular signaling and junctional remodeling.

In parallel, three primary downregulated transcriptional modules were identified, comprising (1) cell-state and developmental transcription factors, (2) mitochondrial bioenergetic and electron-transport programs, and (3) basal translational machinery. Rather than reflecting widespread toxicity or uncoordinated stress, this inverse transcriptional pattern is consistent with a coordinated re-allocation of transcriptional resources, reducing investment in protein synthesis and mitochondrial bioenergetic programs while increasing emphasis on barrier-associated functions, metal homeostasis, xenobiotic handling, and adaptive stress responses.

Figure 5 integrates these gene-level and pathway-level signatures into a unified, transcriptome-informed model illustrating the early cellular response to Burdock root extract.

### 4.6. Strengths, Limitations, and Future Directions

A strength of the study is the integration of complementary analytical approaches, combining gene-level differential expression, pathway-level analyses, and comparator extracts to distinguish shared botanical responses from Burdockpreferential effects. Consistent treatment-group separation across biological replicates and the use of 4.5 h exposure further supported robust characterization of the initial transcriptional response.

The study was conducted in a single keratinocyte cell line; however, the use of a standardized culture allowed the effects of Burdock root extract to be evaluated under controlled and reproducible experimental conditions. All biological replicates were maintained under identical culture and treatment conditions, minimizing environmental variability within the experiment.

The main limitations of the study include the evaluation of only a single treatment concentration and the reliance on transcriptomic data alone for mechanistic interpretation. Accordingly, the findings should be considered hypothesis-generating.

Future studies incorporating proteomic, metabolomic, and functional analyses, together with more physiologically relevant skin models, will help define how these early transcriptional changes translate into cellular function and identify the Burdock root extract constituents responsible for the observed responses.

## 5. Conclusions

Burdock root extract induces a coordinated early transcriptional adaptation in HaCaT keratinocytes, encompassing cytoprotective signaling, metal homeostasis, lipid remodeling, and structural adaptation. Upregulation of NRF2- and AhR-associated genes occurred alongside metallothioneins (*MT1G*, *MT1F*) and zinc transporters, identifying heavy-metal buffering and trace-element regulation as prominent features of the response.

A central axis of this adaptation involved complementary sterol and sphingolipid remodeling. Upregulation of sterol-transport transcripts (*ABCG1*, *OSBP2*, *OSBP*) coincided with the coordinated suppression of de novo cholesterol- and lanosterol-biosynthetic pathways. Concurrently, altered expression of *SPHK1*, *SGPP1*, and *SGPL1* indicated altered transcriptional control of S1P turnover, supporting a model of reduced biosynthetic investment coupled with enhanced sterol transport and sphingolipid recycling. These metabolic shifts occurred alongside coordinated regulation of tight-junction, desmosomal, Hippo-, Rho GTPase-, and TGF-β-associated pathways, consistent with an integrated epithelial adaptation program.

Comparative transcriptomics demonstrated that the Burdock response is distinguished by the magnitude and combination of multiple transcriptional programs rather than by a single isolated pathway. A distinct gene subgroup, including *PMEPA1*, *TGFB2*, *F3*, and *SCARA5*, exhibited stronger regulation in response to Burdock than to Rosehip or Schisandra, whereas *SGPP1* represented a shared botanical response expressed with higher magnitude.

Overall, these findings establish a transcriptome-informed model of early cellular adaptation. Because sterol homeostasis and S1P metabolism involve conserved regulatory pathways across multiple tissues, transcripts such as *ABCG1*, *OSBP2*, and *SGPP1* provide relevant candidate targets for future investigation in intestinal and hepatic models of nutritional intervention and lipid metabolism. At the same time, the coordinated regulation of lipid-metabolic, metal-homeostasis, and epithelial-remodeling pathways highlights biological processes that are directly relevant to epidermal homeostasis and barrier maintenance. Further functional, proteomic, and metabolomic studies will be required to determine whether these gene-level shifts translate into measurable effects on lipid metabolism, skin barrier function, or metabolic regulation.

## Figures and Tables

**Figure 1:**
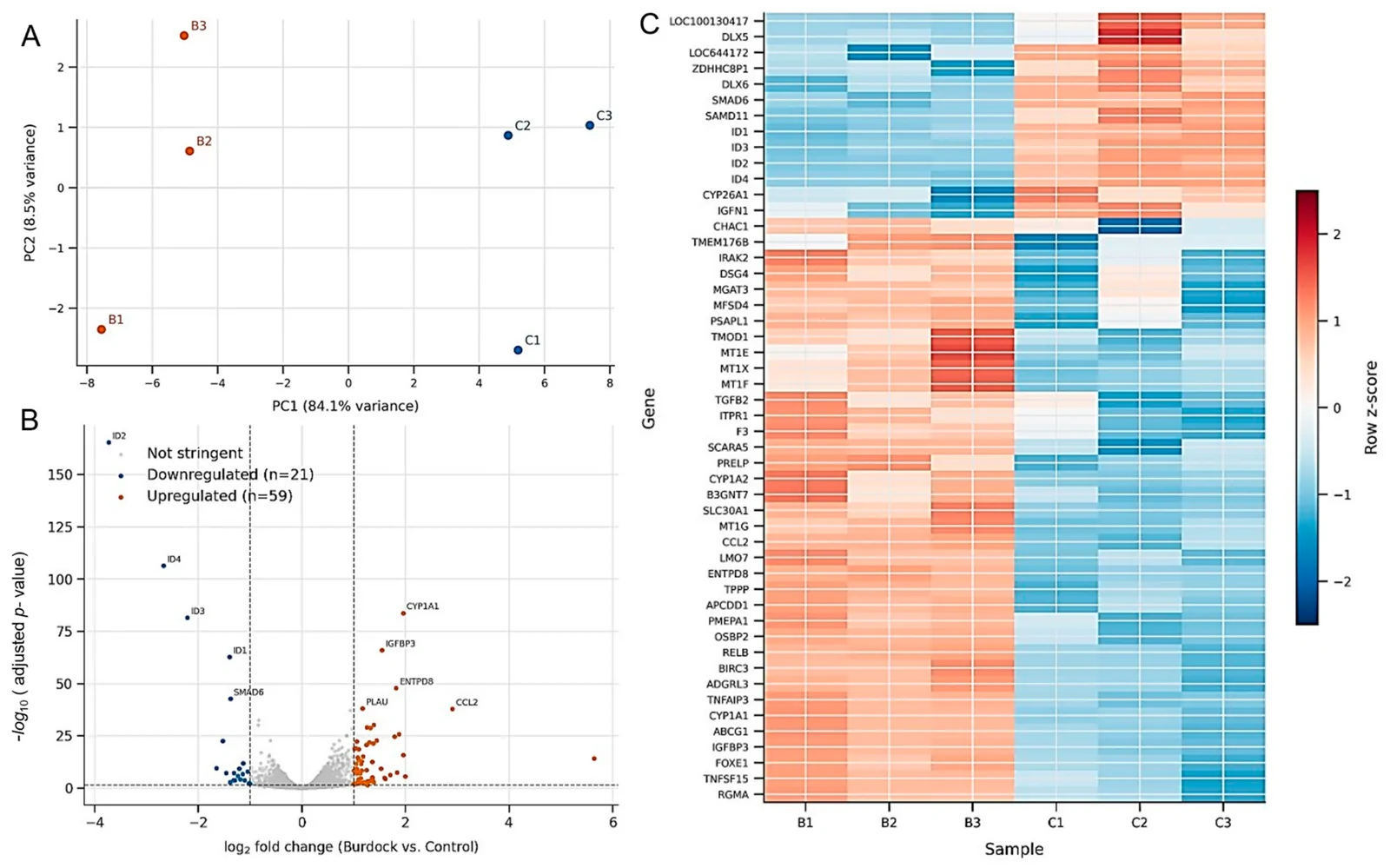
Global transcriptomic response of HaCaT keratinocytes to Burdock root extract. (**A**) PCA of Burdock-treated (B1–B3) and untreated-control (C1–C3) samples based on VST expression values for the 500 most variable genes. (**B**) Volcano plot of the Burdock-versus-control DE analysis. Genes meeting FDR ≤ 0.05 and |log_2_ FC| ≥ 1 are shown as higher-amplitude DEGs, comprising 59 upregulated and 21 downregulated genes. Genes meeting FDR ≤ 0.05, but not the effect-size criterion, are displayed separately. The vertical dashed lines mark the thresholds for the log2 fold change while the horizontal dashed line marks the threshold for statistical significance (based on the adjusted *p*-value). (**C**) Heatmap of the 50 most variable significant DEGs between Burdock-treated (B1–B3) and untreated-control (C1–C3) samples. VST expression values were standardized by gene using z-scores and hierarchically clustered using Euclidean distance and average linkage.

**Figure 2:**
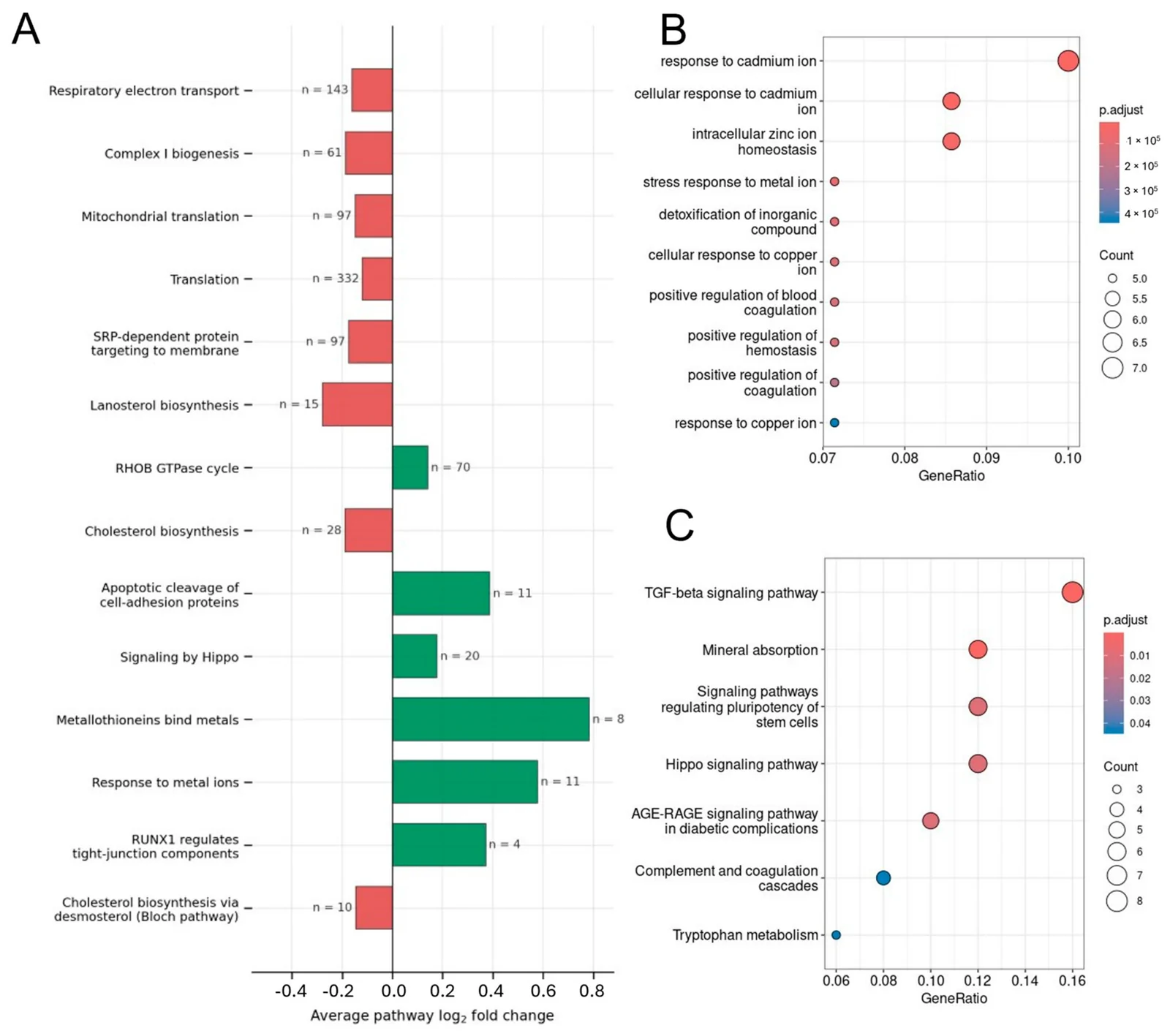
Functional enrichment and directional pathway analysis of the Burdock-versus-control transcriptional response. (**A**) Selected ReactomeGSA pathways meeting FDR ≤ 0.05. Bars represent average pathway log_2_ FC. Negative (in red) and positive (in green) values indicate pathways with negative and positive average directions, respectively. Labels indicate the number of mapped genes contributing to each pathway. (**B**) Focused GO Biological Process ORA of the 80 higher-amplitude DEGs, showing selected terms associated with metal-ion responses, intracellular zinc-ion homeostasis, detoxification of inorganic compounds, coagulation, and hemostasis. (**C**) KEGG ORA of the 80 higher-amplitude DEGs, showing selected enriched pathways. In panels (**B**,**C**), point size indicates the number of mapped input genes, point color indicates FDR, and GeneRatio represents the proportion of mapped input DEGs assigned to each category.

**Figure 3:**
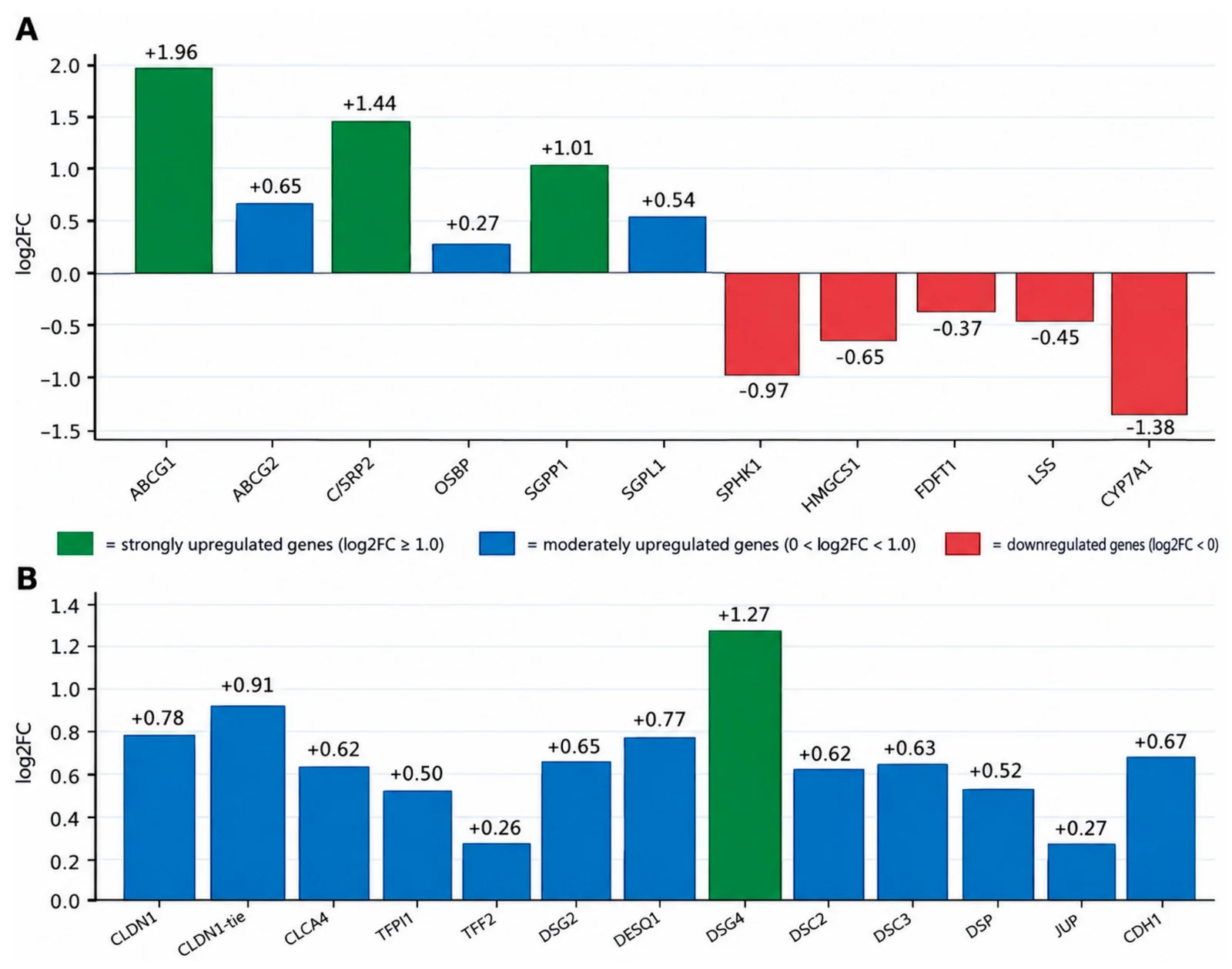
Differential expression of selected lipid-metabolism- and junction-associated genes in the Burdock-versus-control comparison. (**A**) Selected genes associated with sterol transport, cholesterol biosynthesis, retinoid metabolism, and S1P metabolism. (**B**) Selected genes associated with tight junctions, desmosomes, and adherens junctions. Bars represent log_2_ FC values for significant DEGs.

**Figure 4:**
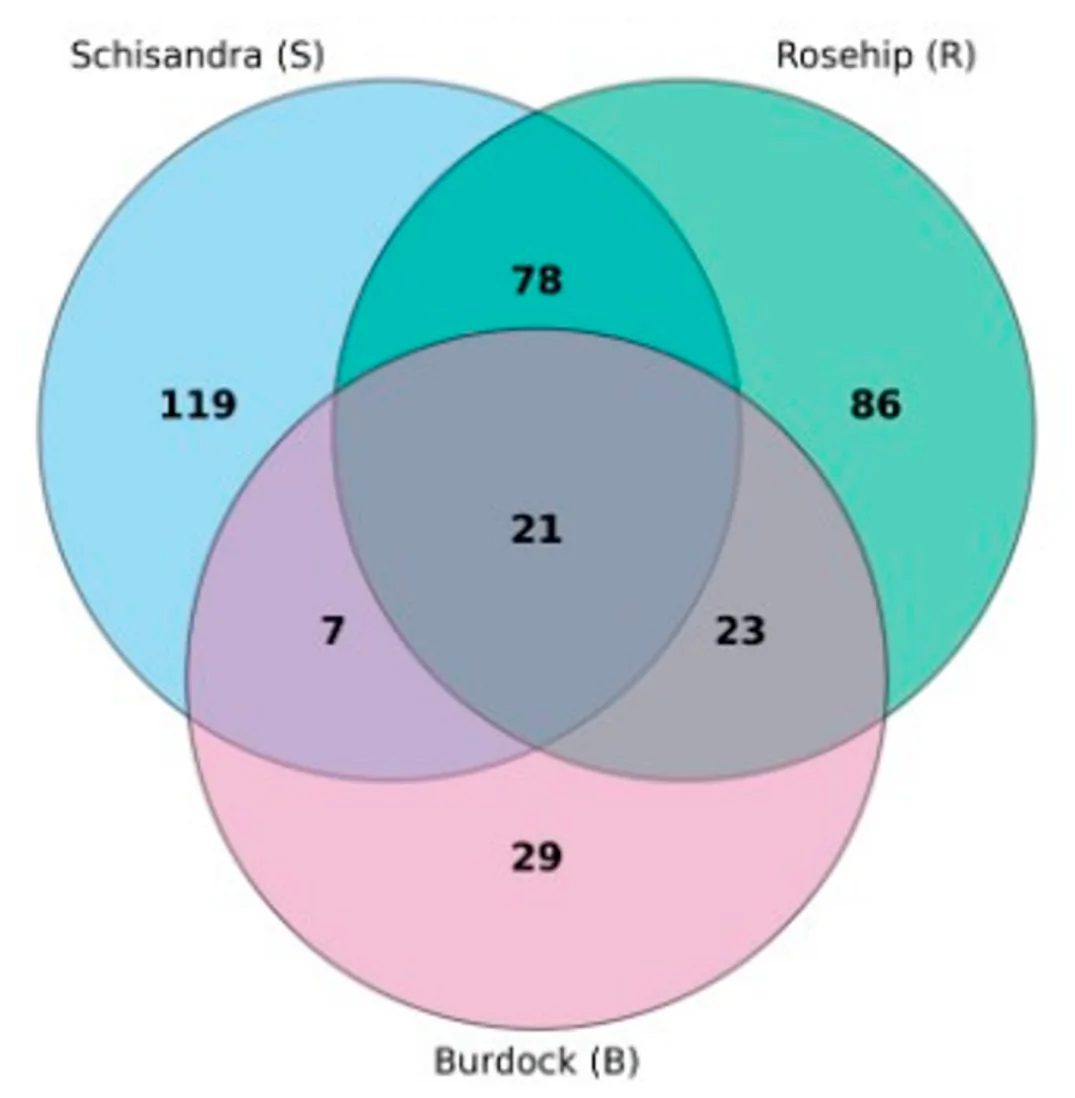
Overlap of higher-amplitude DEGs among Burdock-, Rosehip-, and Schisandra-treated keratinocytes. The Venn diagram shows genes meeting FDR ≤ 0.05 and |log_2_ FC| ≥ 1 in each treatment-versus-control comparison.

**Figure 5:**
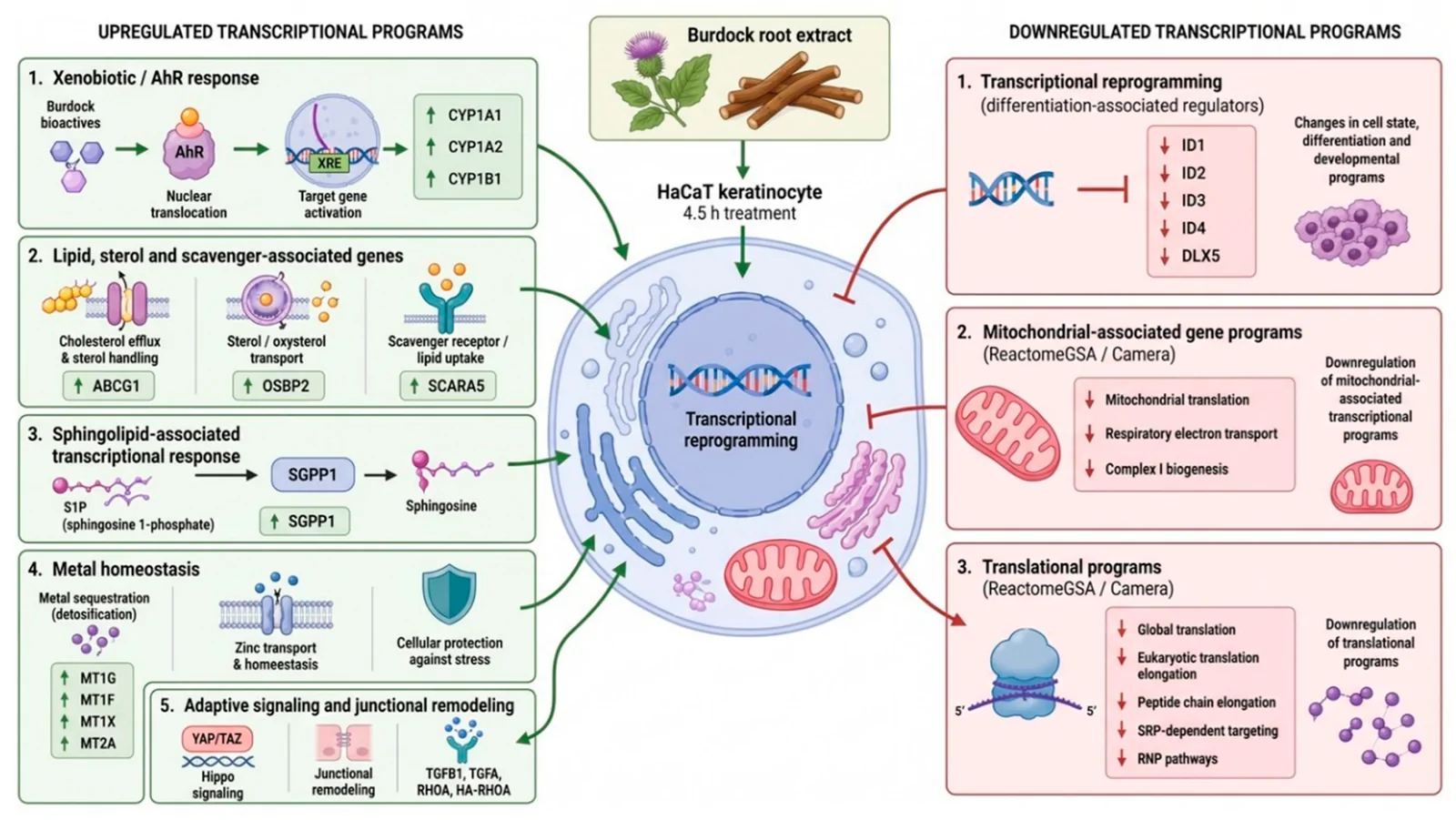
Transcriptome-informed model of the integrated early response of HaCaT keratinocytes to Burdock root extract. Following 4.5 h exposure to Burdock root extract, HaCaT keratinocytes exhibited coordinated transcriptional responses involving AhR-associated xenobiotic defence, metal homeostasis, sterol and lipid handling, sphingolipid-associated regulation, and adaptive signaling pathways linked to pericellular remodeling and junctional organization. Concurrent downregulation was observed in cell-state regulatory networks, mitochondrial bioenergetic programs, and translational machinery. Together, these transcriptomic signatures are consistent with a coordinated early adaptive response characterized by increased investment in stress handling, barrier-associated functions, and cellular homeostasis alongside reduced representation of bioenergetic and protein-synthesis programs. Representative genes and pathways are shown for visualization purposes and summarize significant findings obtained from differential-expression (FDR ≤ 0.05; |log_2_ FC| ≥ 1) and pathway-enrichment analyses (FDR ≤ 0.05), the green and red arrows represent up- and downregulation, respectivelyBiological interpretations extending beyond transcriptomic observations should be regarded as hypothesis-generating, as they were not directly evaluated using proteomic, metabolomic, or functional assays. Created in https://BioRender.com.

**Table 1:** Sequencing depth and differential-expression summary of the Burdock extract versus control comparison. Raw-read counts represent reads produced by sequencing before quality trimming. Retained-read counts and unique-alignment rates were obtained from the aggregated MultiQC report following sequence preprocessing.

Metric	Burdock Treated Sample	Untreated Control
Biological replicates, n	3	3
Raw reads/sample, million	124.52–144.18	129.21–156.50
Mean raw reads/sample, million	136.84	140.78
Reads retained after preprocessing, million	120.9–140.5	123.6–151.1
Unique alignment rate, %	94.1–94.3	93.1–93.5
Significant DEGs, FDR≤0.05	4044	Not applicable
Upregulated/downregulated DEGs, FDR ≤ 0.05	2052/1992	Not applicable
Higher-amplitude DEGs, FDR≤0.05 and |log2 FC| ≥ 1	80 total, 59 upregulated and 21 downregulated	Not applicable

**Table 2:** Burdockextract responsive genes not significantly expressed in either comparator analysis. Genes met FDR ≤ 0.05 and |log_2_ FC| ≥ 1 in the Burdock-versus-control comparison but had FDR > 0.05 in both comparator-versus-control analyses. Functional annotations provide concise biological context.

Gene	log_2_ FC, Burdock vs. Control	FDR	Concise Functional Annotation
*JUN*	+1.048	4.94 × 10^−10^	AP-1 transcription factor involved in the regulation of cellular stress, inflammatory, proliferative, and differentiation-associated responses.
*OSBP2*	+1.439	1.61 × 10^−23^	Intracellular sterol binding and transport, with potential involvement in membrane-lipid organization.
*PMEPA1*	+1.836	4.22 × 10^−8^	TGF-β-responsive transmembrane regulator involved in negative feedback of TGF-β signaling, receptor turnover, and cell-growth regulation.
*B3GNT7*	+1.359	1.13 × 10^−5^	Glycosyltransferase involved in poly-N-acetyllactosamine extension and organization of cell-surface glycans.
*F3*	+1.113	2.35 × 10^−3^	Tissue factor involved in coagulation-associated signaling and potentially related to inflammation, migration, and pericellularmatrix responses.
*MT1G*	+5.637	7.86 × 10^−15^	Intracellular metal-ion binding, zinc buffering, and oxidative-stress adaptation.
*TGFB2*	+1.021	1.81 × 10^−2^	TGF-β-family cytokine involved in differentiation, extracellular-matrix remodeling, and tissue-response signaling.
*SCARA5*	+1.589	1.39 × 10^−5^	Scavenger receptor involved in ferritin recognition and cellular iron handling.
*MT1F*	+1.615	4.00 × 10^−5^	Metal-ion binding and protection against metal-associated oxidative stress.
*TMEM176B*	+1.190	2.02 × 10^−3^	Intracellular cation-channel protein associated with and immune-regulatory and cellular signaling processes.
*SCARNA2*	−1.104	2.41 × 10^−4^	Non-coding RNA involved in RNA processing and DNA-repair regulation.

## Data Availability

The raw RNA-sequencing data and associated analysis code are owned by Finzelberg GmbH & Co. KG and are not publicly available because they were accessed under a Confidential Disclosure Agreement. Requests for access may be directed to Dr. Cynthia G. Suarez and will be evaluated by Finzelberg GmbH & Co. KG, subject to the applicable confidentiality conditions.

## Supplementary Materials

The following supporting information can be downloaded at https://www.mdpi.com/article/10.3390/metabo16100707/s1: Table S1. Physicochemical characterization of the aqueous extract of burdock roots (TPA 39-22-1); Figure S1. Thin-layer chromatographic profile of the aqueous burdock root extract (lane 1) and reference standards (lane 2). TLC plates were visualized under UV light (365 nm) after derivatization with diphenylboric acid aminoethyl ester and macrogol reagents. The labels in the reference lane indicate caffeic acid (Kaffeesäure R), hyperoside (Hyperosid R), chlorogenic acid (Chlorogensäure R), and rutin (Rutosid R); Figure S2. Sample-distance matrix for the complete experimental dataset. Distances among untreated-control (C1–C3), Burdock- (B1–B3), Rosehip- (R1–R3), and Schisandra-(S1–S3) treated samples were calculated from VST expression values across all 12 biological samples.
