# Supplementary material for: Burdock (Arctium lappa L.) Root Extract Induces Transcriptional Signatures Associated with Metal Homeostasis and Lipid Metabolism in Human Keratinocytes
Source: Metabolites. 2026 Sep 24;16(10):707. doi: 10.3390/metabo16100707 (PMC13707041; doi:10.3390/metabo16100707)
Supplement: Supplementary file 1 [file metabolites-16-00707-s001.zip › metabolites-4559149-supplementary.pdf]

Table S1: Physicochemical characterization of the aqueous extract of burdock roots (TPA 39-22-1)

Physical Parameters	Results
Water content [1]	3.5 %
Ash content [1]	5.4 %
Bulk density [1]	809 g/L
**Phytochemical Parameters**	
Phytochemical profile by TLC [2]	See Figure S1
Caffeoylquinic acids by HPLC [3]	1.9 %
Total polyphenols by UV-VIS [1]	3.7 %
Total flavonoids by UV-VIS [1]	Not detected
**Contaminants**	
Aflatoxins [4]	
Aflatoxin B1	0.1 ppb
Total aflatoxins (B1 + B2 + G1 + G2)	0.35 ppb
Heavy Metals [4]	
Lead	0.06 ppm
Cadmium	0.04 ppm
Mercury	< 0.01 ppm
Arsenic	0.22 ppm
Pesticides [4] (analysis covering more than 370 substances)	compliant
Microbiology[1]	
Total aerobic microbial count (TAMC)	< 100 CFU/g
Total combined yeasts/moulds count (TYMC)	< 10 CFU/g
Bile-tolerant gram-negative bacteria	< 1 CFU/g
Pseudomonas aeruginosa	Absent in 1 g
Escherichia coli	Absent in 1 g
Staphylococcus aureus	Absent in 1 g
Salmonella spp.	Absent in 25 g

[1] Methods according to European Pharmacopeia.

[2] The phytochemical profile was determined by thin-layer chromatography (TLC). The extract was dissolved in methanol : water (70:30, v/v), applied to a silica gel F254 plate, developed using formic acid/water/ethyl acetate as the mobile phase, derivatized with diphenylboric acid aminoethyl ester and macrogol reagents, and examined under UV light (365 nm). A reference solution containing rutin, chlorogenic acid (CGA), hyperoside, and caffeic acid was used.

[3] Quantification of caffeoylquinic acids (CQAs) was performed by HPLC-UV on a C18 column (250 × 4.6 mm, 5 µm particle size) maintained at 40 °C. The mobile phase consisted of acetonitrile : phosphoric acid : water (70:4:926, v/v) (solvent A) and acetonitrile : phosphoric acid (996:4, v/v) (solvent B). The flow rate was 1.5 mL/min, detection wavelength 330 nm, and injection volume 10 µL. A CGA reference standard was dissolved in methanol : water (70:30, v/v) to obtain a concentration of 70 µg/mL. Results were calculated from the ratio between sample peak areas and the CGA calibration solution and expressed as percentage CQAs, calculated as CGA equivalents in the extract.

[4]Analysis conducted by accredited, specialized laboratory.

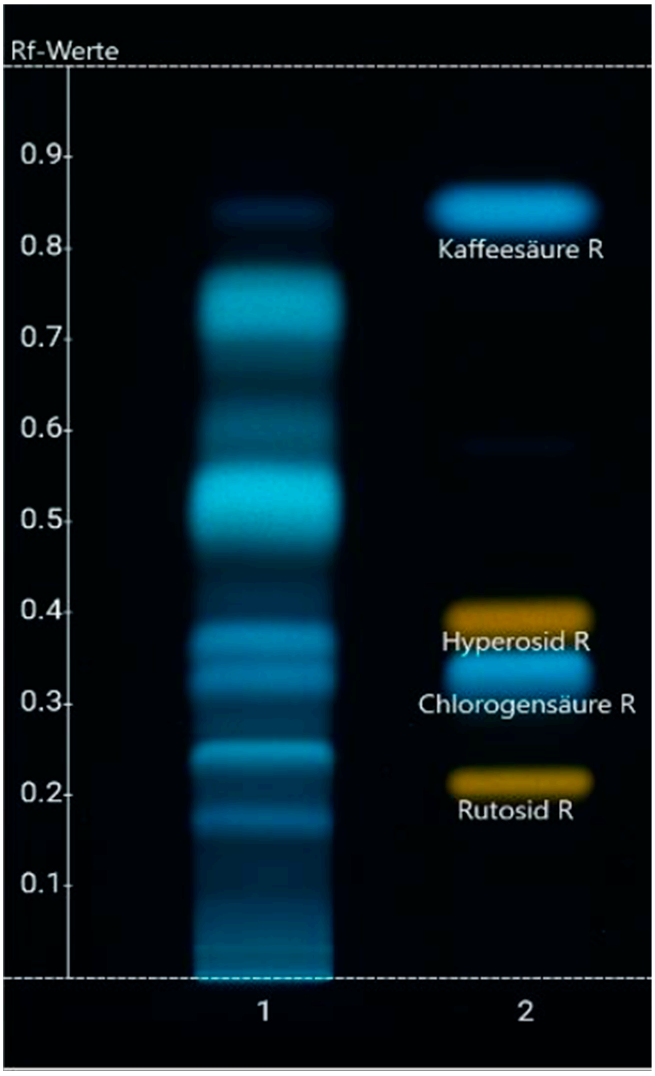

Figure S1. **Thin-layer chromatographic (TLC) profile of the aqueous burdock root extract (lane 1) and reference standards (lane 2).** TLC plates were visualized under UV light (365 nm) after derivatization with diphenylboric acid aminoethyl ester and macrogol reagents. The labels in the reference lane indicate caffeic acid (Kaffeesäure R), hyperoside (Hyperosid R), chlorogenic acid (Chlorogensäure R), and rutin (Rutosid R).

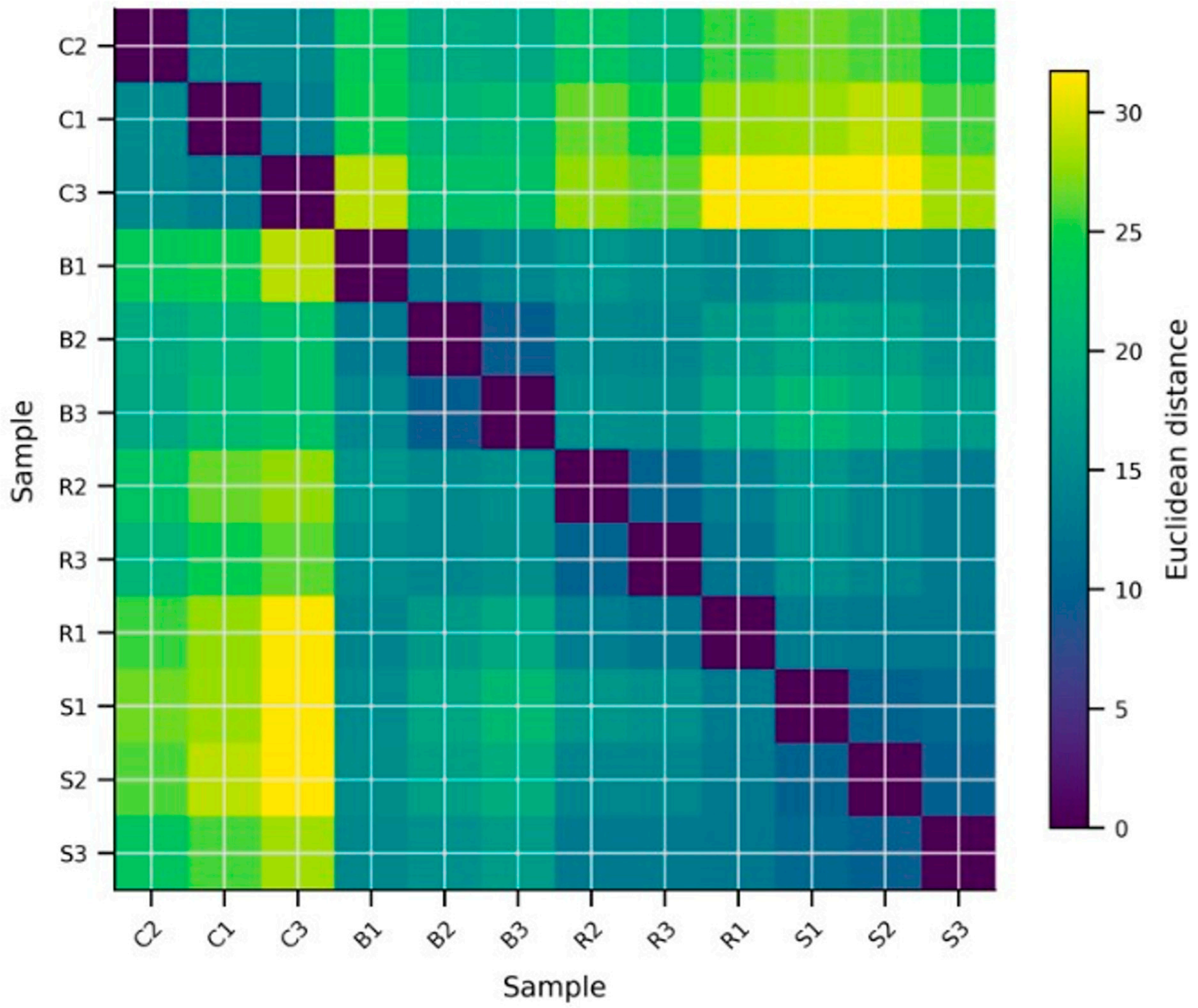

Figure S2. Sample-distance matrix for the complete experimental dataset. Distances among untreated-control (C1-C3), Burdock- (B1-B3), Rosehip- (R1-R3), and Schisandra-(S1-S3) treated samples were calculated from VST expression values across all 12 biological samples.
